# A central role for Galectin 3 during renal epithelial cell morphogenesis after nephrectomy

**DOI:** 10.1186/2046-2530-1-S1-P76

**Published:** 2012-11-16

**Authors:** F Poirier, A Viau, J Magescas, M Burtin, F Terzi, D Delacour

**Affiliations:** 1 Institut Jacques Monod CNRS Paris Diderot, France; 2 INSERM U845, France

## 

Galectin-3, a member of the multigene family of beta-galactoside binding lectins, is mainly expressed in epithelial cells. The distribution of galectin-3 depends on cell type and cell differentiation. We previously showed that galectin-3 is transiently associated with the centrosome, and more specifically with the basal body at the basis of the primary cilium of MDCK cells. In the present study, we use *galectin-3* null (*Gal3-/-*) mutant mice to investigate the functional consequences of our observations in the context of kidney regeneration after subtotal nephrectomy. Three months after surgery, renal functions were more severely affected in *Gal3-/-* mutant than in wt animals. Upon sacrifice, we found that *Gal3-/-* mutant kidneys were bigger and heavier, and this hypertrophy was associated with large tubular dilatations. Interestingly, in wt mice, galectin 3 intracellular distribution was altered in response to injury, displaying a distinct centrosomal localization in epithelial cells lining the collecting ducts close to the wound, whereas in cells away from the wound galectin-3 remained uniformly cytosolic. The impact of nephron reduction on primary cilium growth was examined by 3D reconstruction of wt and *Gal3-/-* renal tubules stained for acetylated alpha-tubulin. This analysis revealed that mutant primary cilia are irregular, bent, twisted and longer than wt primary cilia, indicating that Galectin-3 is essential for primary cilium growth. This suggests that the failure in kidney regeneration observed in *Gal3-/-* mutant mice may be due to defects in primary cilium biogenesis.

